# Resilience of informal settlements to climate change in the mountainous areas of Konso, Ethiopia and QwaQwa, South Africa

**DOI:** 10.4102/jamba.v12i1.778

**Published:** 2020-03-12

**Authors:** Tamirat W. Melore, Verna Nel

**Affiliations:** 1 Department of Urban and Regional Planning, University of the Free State, Bloemfontein, South Africa

**Keywords:** climate change risks, systems approach, resilience, indigenous knowledge, informal settlements

## Abstract

Managing change is essential for human survival; thus, the importance of adapting to climate change has been increasingly recognised by researchers and governments alike. This is reflected in the growing literature on climate change and the imperative for action including building resilience in our socio-ecological systems. Despite the large body of research that now exists, few studies have considered the resilience of informal rural or peri-urban settlements in mountainous regions. This article considered the resilience of two rural settlements in mountainous areas, namely Konso, Ethiopia, and QwaQwa, South Africa, to the influences of climate change based on the assets available to them. The authors obtained the local communities’ perception of their risks throsugh interviews with community leaders and a survey of 384 residents, divided equally between each settlement. Furthermore, the resilience of each community was assessed on the basis of their environmental, social, economic, human, institutional and physical capitals using a climate change resilience indicator. The findings showed that both communities faced major challenges because of climate change, particularly from drought and poverty. We found that both communities retained some forms of indigenous knowledge, but its greater application in Konso appeared to improve resilience to a greater extent than QwaQwa, where it played a lesser role and the community was more dependent on the government. However, indigenous knowledge alone is not sufficient to support these communities in the long term, given the growing aridity of the regions, and other approaches are also necessary, including government support, to enhance and grow their capitals.

## Introduction

Climate change is affecting large parts of the world with more destructive and frequent hurricanes and typhoons, melting ice caps and the warming of the oceans along with devastating droughts and floods (Bai et al. 2018). The nature and intensity of climate change events and risks differ from place to place, but the impact on poorer countries and communities is particularly severe. Informal settlements in developing countries, in general, and Africa, in particular, have heightened risks associated with climate change (United Nations International Strategy for Disaster Reduction [UNISDR] 2011:1–7).

Africa has experienced rapid population growth along with urbanisation. It is anticipated that the population will increase from about 1 billion in 2010 to nearly twice that by 2040 (UN-Habitat 2014:17). One of the push factors for urbanisation is the degradation of the natural environment and low agricultural productivity because of climate-change-induced risks, while natural increase also contributes to the burgeoning population (Fox 2014:257–283). Not only the larger settlements but also the smaller towns and villages are increasingly vulnerable to political, economic and social risks exacerbated by climate change. These factors, along with chronic poverty, threaten the living conditions of poor people and increase the complexity of the challenges they face (Dodman et al. 2015:5). ‘The combination of demographic pressures, rapid urbanisation, environmental and climate change now appear to reinforce a host of negative urban externalities’ (UN-Habitat 2014:7). In general, the poor are more susceptible to the consequences of climate change threats; this is because of limited resources they have that are necessary to cope with the adverse impacts (Adelekan et al. 2015; Mearns & Norton 2010:5).

In many cases, migrants from rural areas are compelled to live in informal settlements on the periphery of urban areas, often in hazardous spaces, such as flood-prone areas and steep hillsides. With their high levels of poverty, inequality and informality, it is unsurprising that South Africa and Ethiopia were categorised, respectively, as medium-level and very high-level risks in terms of the vulnerability of their population to climate change (United Nations Universities, Institute for Environment and Human Security 2011).

Resilience to various threats and risks, including climate change and its impacts, has generated a large body of research (Jiang et al. 2017:2224; Martin-Breen & Anderies 2011:12–55; The Royal Society 2010). Both the resilience and climate change literature cover a range of topics and regions, including the cities of the North and the rural regions and shanty towns of the Global South. However, only a few studies have considered the resilience of informal rural or peri-urban settlements in mountainous regions (e.g. Barua et al. 2014; Belay et al. 2017:812–823; Mekuyie, Jordaan & Melka 2018:64–77). Yet, the characteristics of many small towns in hilly or mountainous areas of Africa require policy-makers to also consider these settlements in developing resilience to climate change. Additionally, they need to consider the assets and wisdom, including indigenous knowledge, available to such communities, and how to build on them to manage, mitigate and adapt to climate change.

Our article examines the role of various capitals, including indigenous knowledge, in building resilience to climate change in two mountainous areas: one in South Africa and the other in Ethiopia. Both areas lie at a similar elevation (approximately 1650 m above mean seal level) and are part of United Nations Educational, Scientific and Cultural Organisation (UNESCO) World Heritage areas. They face similar threats of drought and intense weather events and have developed indigenous knowledge systems to deal with these threats. As one author had resided in both regions, he could gain access to the communities to conduct the research. The case studies are from informal settlements found at the periphery of traditional small towns, specifically at the peripheries of Phuthaditjhaba (QwaQwa, South Africa) and Karat (Konso, Ethiopia).

The findings showed that both communities faced major challenges because of climate change, particularly from drought and poverty. While both communities retained some forms of indigenous knowledge, its greater application in Konso appeared to improve resilience to a greater extent than QwaQwa. However, indigenous knowledge alone is not sufficient to support these communities in the long term, given the growing aridity of the regions, and other approaches are also necessary.

In the next section, we consider key concepts concerning resilience, assets and capitals as well as indigenous knowledge as a form of human capital. Thereafter, we describe our methodology and findings before discussing the implications and conclusions.

## Resilience capacity and indigenous knowledge

Human settlements, from cities to rural villages, are all socio-ecological systems (SES) (Du Plessis 2008:3) in which human activities have direct or indirect influence on the ecological system. Building long-term resilience requires understanding the interdependencies between socio-economic systems and the natural environment, and how human activities bolster or erode the capacity of the greater system to function effectively (Walker & Salt 2006). Understanding urban and rural settlements as an SES requires a dynamic interpretation of resilience that accepts uncertainty, change and adaption (Seeliger & Turok 2013:2108–2128). Much of the literature on resilience stresses the ecological component of SES and environmental maintenance or improvement (cf. Elmqvist 2014:26–30; Folke et al. 2010:20–28; Gunderson 2000; Martin-Breen & Anderies 2011).

However, in this article, we focus on the social component of the SES and resilience capacity of specific places, particularly the assets or capabilities that humans have in order to deal with change, accepting that the resilience capacity of a certain area is reliant on the potential of these assets or capitals (Tinch et al. 2015:323–337). According to Pigg et al. (2013:492–502), the capitals interact and mutually reinforce each other. The number of capitals identified varies within the literature (Emery & Flora 2009:19–35; Tinch et al. 2015:323–337). For the purpose of this research, we adopted the six capitals suggested by Cutter, Ash and Emrich (2014:65–77), namely social, human, economic, physical, institutional and natural capital. This is in line with many other resilience indicators based on a capitals framework (Shipper & Langston 2015:11). Each of these is elucidated below.

*Social capital* comprises social networks that may include communication channels, relatives, community-based voluntary organisations, schools and trade unions, as well as cultural and social norms. It encompasses different formal and informal organisational arrangements people use to work towards shared visions. The trust and synergy within society not only create an opportunity to reduce the costs of production but also create informal safety nets for the poorer section of society (Simone 2001:102–117). Social capital in the form of networks is particularly important in coping with disasters or resolving collective problems. Resilience is enhanced where communities work together towards common goals (Davidson 2006:35).

*Human capital* pertains to the ability to perform productive work. Additionally, the quality of the labour force depends on skills, health, educational status and leadership capacity. Human capital can thus be measured through education, health, population density, population growth, demographic characteristics, access to services, household characteristics, housing quality and dependency ratio (Smith, Simard & Sharp 2001:7–8).

*Economic capital* refers to the ‘financial resources available to invest in community capacity-building, to underwrite the development of businesses, to support civic and social entrepreneurship, and to accumulate wealth for future community development’ (Emery & Flora 2009:19–35). Such financial assets enable households and communities to avert, respond to and rebuild after disasters.

*Physical capital* pertains to the infrastructure available to a community and includes communication and transport infrastructure, housing and other buildings (Emery & Flora 2009:19–35). The presence of physical capital affects the adaptive capacity and resilience of a community (Tinch et al. 2015:323–337).

*Natural capital* refers to those natural resources and ecosystem services essential to sustain life and for the production of other outputs. It includes biological and ecological resources and can be categorised as resources, living systems and ecosystem services (Guerry et al. 2015:7348–7355).

*Institutional capital* includes the social rules, regulations, programmes, policies and governance structures that enable and support resilience capacity in the area. Building such resilience is dependent on the degree of coordination and collaboration among stakeholders (e.g. government, private sector and civil society organisations) (Andersson & Ostrom 2008:71–93).

One element of social, human and institutional capital is indigenous or traditional knowledge that local communities have and use, thus constituting the ‘sum of knowledge and skills constitutive of their meaning, belief systems and dynamic livelihood constructions that distinguish them from other groups’ (A-Magid 2011:136–148). Such knowledge is unique for each community and is transferred from one generation to the other, usually by oral traditions and cultural rituals (World Bank 1998). As it is peculiar to their habitat, it is thus valuable for agricultural activities as well as for maintaining biodiversity. Using this knowledge, farmers are able to cultivate the most appropriate plants and to predict and prepare for adverse weather (Nyong, Adesina & Elasha 2007:787–797). It enables communities to respond collectively to hazards and promote resilience capacity of the SES as they continually accumulate experiences that enable them to cope with crises.

Resilience theory also recognises that indigenous knowledge transferred over a long period assists communities in responding to disaster risks and improving their capacity to respond to shocks (Berkes, Colding & Folke 2000:1251–1262). Consequently, indigenous knowledge is a fundamental capital for the rural poor, enabling contextual solutions for local challenges. The combination of traditional and western scientific knowledge holds the promise of new frames of knowing that are complementary, which can bridge the differences between world views and cultures (Bohensky & Maru 2011:6). Furthermore, integrating indigenous knowledge with modern technology-based science and enhancing the capacity of traditional institutions can enable communities to cope better with the danger of loss of indigenous knowledge in the current generation. One of the critical challenges is transferring and documenting this knowledge to future generations (Nyong et al. 2007:787–797) as it is being eroded by globalisation (A-Magid 2011:136–148).

Accepting traditional and indigenous knowledge recognises other forms of knowing as equally valid. Recognising alternative forms of knowledge is essential when planning in complex systems where flows, activities and relationships co-exist in dynamic interactions with each other, and a relational perspective of space and time is necessary (Healey 2007:224). Consequently, modernist, scientific perspectives cannot be privileged over other interpretations or meanings in an SES as they are only one component of the multiple experiences, associations and understandings of the communities within the system (Baskin 2008:1–12; Innes & Booher 2015:195–213).

## Method

Our research used a comparative case study research design and adopted multiple sources of data and methods of analysis (Creswell & Clark 2017:4; De Vos et al. 2005:66; Rule & John 2011:61). Climate data (precipitation and temperature) were collected from both South African and Ethiopian meteorology agencies to identify the trends over the past 30 years. Additional secondary data were obtained from socio-economic maps, satellite images, spatial development plans and official publications (Agricultural Research Council Institute for Soil Climate and Water [ARCISCW] 2016; National Meteorology Institute of Ethiopia [NMIE] 2015).

Primary data were gathered from officials, community leaders and residents in the two study areas using a combination of data collection tools. Semi-structured interviews were conducted with 18 purposively selected respondents such as municipal officials, community leaders and traditional leaders (nine respondents per study area). Furthermore, semi-structured questionnaires were distributed to residents in each study area.

Each of the study sites housed 5000 households living informally on the periphery of each town (Konso Municipality 2014:42; Maluti-a-Phofung Local Municipality 2018). These informal settlements were each divided into four regions, and then using a simple random sampling method (Kothari 2004:178), a sample of 182 households per region and 48 households per cluster was selected for the administration of the questionnaires to one per household.

Respondents were purposively selected for the interviews based on their knowledge and position in the community (government officials, municipal councillors, school principals and church pastors as well as one non-governmental organisation (NGO) per study area). In addition to these respondents, three traditional leaders per study area were identified through snowball sampling. Although 26 people approached, only nine semi-structured interviews per region were conducted (18 in total). Furthermore, a 3-hour group discussion was held with the nine respondents who had been interviewed in each study area with the aim of triangulating and enhancing the validity of data captured from the semi-structured interviews.

The raw data were transformed into percentages, rates or averages to enable comparisons. Based on the surveys, a comparison of the respondents’ perception on the risks of climate change and weather events was performed, as indicated in Tables 1 and 2.

**TABLE 1:** Phuthaditjhaba respondents’ perceptions of climate-change-induced risks.

Climate-change-induced risks	Intensity of risk (%) (*n* = 205)		
	High	Medium	Low
Drought or water shortages	100	0	0
Severe thunderstorms	95	5	0
Flash flooding	85	15	0
Strong damaging winds	85	15	0
Very cold winters	75	25	0
Very hot summers	65	35	0
Bush (veld) fires	55	45	0
Loss of biodiversity	95	5	0
Soil erosion	100	0	0
Landslides or rock falls	90	10	0
Loss of grazing land	90	10	0
Decrease in soil fertility	90	10	0

**TABLE 2:** Karat respondents’ perceptions of climate-change-induced risks.

Perceived risks	Intensity of risk (%) (*n* = 205)		
	High	Medium	Low
Drought or water shortages	100	-	-
Food shortages	81	-	-
Reduced agricultural productivity	100	-	-
Prevalence of tropical diseases (e.g. malaria)	98	-	-
Heat waves	82	-	-
Loss of biodiversity	95	-	-
Flash flooding	-	62	38
Bush (veld) fires	-	12	88
Strong damaging winds	-	-	100
Very cold temperatures	-	-	100
Soil erosion	-	-	87
Landslides or rock falls	-	6	94
Loss of grazing land	-	15	85

To develop a proxy for resilience, a climate change resilience index encompassing social, economic, human, physical, natural and institutional capitals was developed, following the process suggested by Asadzadeh et al. (2017). The resilience index for each of the capitals was calculated on a scale of 0–1. The selection of variables was based on a review of the literature as well as the availability of data (Tyler et al. 2016:432). Social capital variables included number of social networks, safety net programmes and indigenous knowledge, while the economic capital included household income, employment rate, dependency rate, savings and investments, and availability of micro-finance. The human capital variables included life expectancy, education, availability of skilled and unskilled labour, population density and fertility rate. Physical capital focussed on infrastructure such as roads and storm water drainage, housing costs and the costs of terracing and retaining walls. Costs of wood for fuel, availability and access to water, social conservation and the productivity of climate-adapted trees and crops were included among the natural capital indicators. Each indicator was assigned an equal weighting as there was no theoretical justification for a differential weighting. Based on Statistics South Africa (Stats SA) 2011 census data and the Ethiopian Central Statistics Agency 2007 census data, an index for each capital and each area was calculated (see Table 3).

**TABLE 3:** Resilience risk index based on calculations of capitals.

Capitals	Phuthaditjhaba resilience index	Karat resilience index
Social	0.056	0.051
Economic	0.040	0.037
Human	0.031	0.030
Physical	0.028	0.028
Natural	0.056	0.033
Institutional	0.056	0.051
Composite index	0.044	0.038

## Findings

In our discussion of the case study areas, we first provide some background including the anticipated climate change impacts, followed by a discussion of the assets or capitals and the role of indigenous knowledge in each area. Thereafter, we discussed the comparative resilience to climate change of both Phuthaditjhaba, South Africa, and Karat, Ethiopia.

We found that both areas are expected to become drier with more droughts. Neither area is endowed with many assets, with high levels of unemployment and poverty, poor or limited infrastructure and weak institutions. Thus, the effects of climate change are expected to worsen the already fragile livelihoods of the two communities. However, the stronger indigenous knowledge practiced in Karat has protected their natural environment to a greater extent than is the case in Phuthaditjhaba.

### Phuthaditjhaba

**FIGURE 1:**
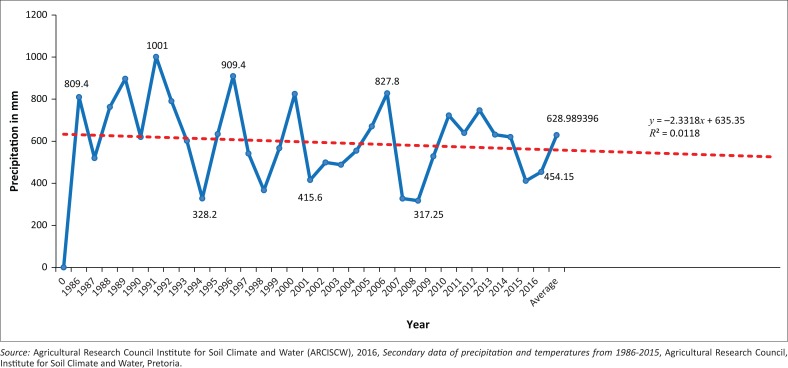
Phuthaditjhaba mean annual precipitation pattern for period 1986–2016 (in mm) and projection up to 2030.

Climate change projections for South Africa forecast drier conditions in the west and more frequent and intense storms in the east of the country. Additionally, the average temperature is expected to rise by 2ºC by the middle of this century (Mearns & Norton 2010:7; UN-Habitat 2014:231–232). The recorded rainfall from 1986 to 2016 is indicted in Figure 1.

According to the respondents, the Phuthaditjhaba area already manifests the impact of climate change with increasing summer temperatures, fluctuations in rainfall leading to periodic severe shortages of water, increased incidences of drought, strong, damaging winds, flash flooding causing soil erosion and rock falls from hillsides, along with a reduced biodiversity. The growth of informal settlements is driven by various factors such as urbanisation, land speculation and the inability of the municipality to meet the demand for land and housing, intensified by previous apartheid policies (ARCISCW 2016).

Municipal officials are not in favour of *in situ* upgrading of informal settlements, preferring demolition and relocation. While some informal settlements may be on hazardous sites, and relocation may be safer, the uncertainty of tenure created by the official’s attitude erodes the community’s resilience. Furthermore, the perceptions and planning approach of the municipality do not take the factors influencing the formation of informal settlements into account. Viewing informal settlements as a constraint to development, they fail to provide basic infrastructure and services in the area, thus depriving the community of physical capital. This reluctance is deepened where the settlements are located on traditional land. The juxtaposition of municipally controlled areas and traditional authority areas along with the overlap of responsibilities between municipal councillors and the traditional authority (especially in the process of land administration) opens the door for conflict (Duma 2016:24).

The conflict of authority between the tribal leaders and the municipality negatively affects development, including environmental conservation. Degradation of the natural environment at the periphery of Phuthaditjhaba because of overgrazing, veld fires and excessive extraction of soils for moulding bricks was observed. This suggests that community has not successfully utilised their social and institutional capital to manage communal resources.

The residents of the Maluti-a-Phofung Municipality, most of whom reside around Phuthaditjhaba, are poor with over two-thirds earning under R1600 per month (Maluti-a-Phofung 2018:23). Many are dependent on social grants. Thus, their economic capacity to cope with climate change risks is low. The World Heritage site status of the Golden Gate National Park, the Drakensberg Mountain range around Phuthaditjhaba and the cultural heritage of the community attract many tourists to the area. The income generated from this tourism sector could be utilised for providing infrastructure, but this opportunity has not been utilised effectively. The lack of infrastructure and service facilities to attract manufacturing and other investments to the area contributes to a high unemployment rate of over 50% and low household incomes (Stats SA 2012:1–41).

The scarcity of all the capitals along with weak coping strategies diminishes their climate change resilience capacities, exacerbating the severity of the risks in the area (see Table 3). Inadequate road layout and drainage systems aggravate the damage caused by storm water runoff. During emergencies, limited open spaces or alternative routes, along with narrow internal roads, can hamper rescue and evacuation operations. Yet, there are no disaster risk reduction measures, preparedness plans, early warning systems and trained manpower in these communities to manage and mitigate risks. The lack of interest of the municipality regarding the needs of the informal settlements also contributes to a lower climate change resilience capacity in the study site. Although the communities can identify risks that they face, they believe there is little that they can without the support of the municipality.

The research revealed that no form of indigenous knowledge is fully and effectively utilised to improve the resilience capacity in the area. Furthermore, there are no solid and well-organised community-based institutions or systems for the recording or extension of indigenous knowledge in the local area. It is thus neither well documented nor institutionalised and is becoming limited to elderly people.

Over 65% of the respondents agreed on the existence of some practices of integration of indigenous and scientific knowledge for natural resource management and the identification of locally adapted crops in the area. Nevertheless, many respondents agreed that integration of indigenous knowledge with scientific knowledge was not observed in practice, including in the urban planning function. The absence of strong local institutions (formal municipal and traditional) that enhance the benefit of indigenous and scientific knowledge to improve climate change resilience capacity of the area is one of the contributing factors for the low overall resilience of the community.

### Karat

**FIGURE 2:**
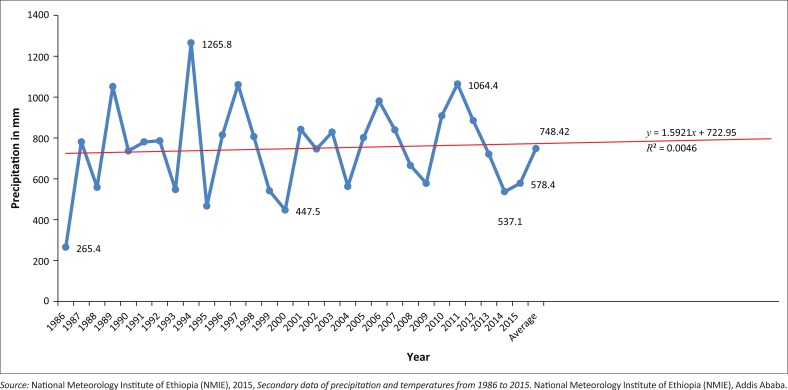
Konso mean annual precipitation pattern for period 1986–2015 (in mm) and projection up to 2030.

**FIGURE 3:**
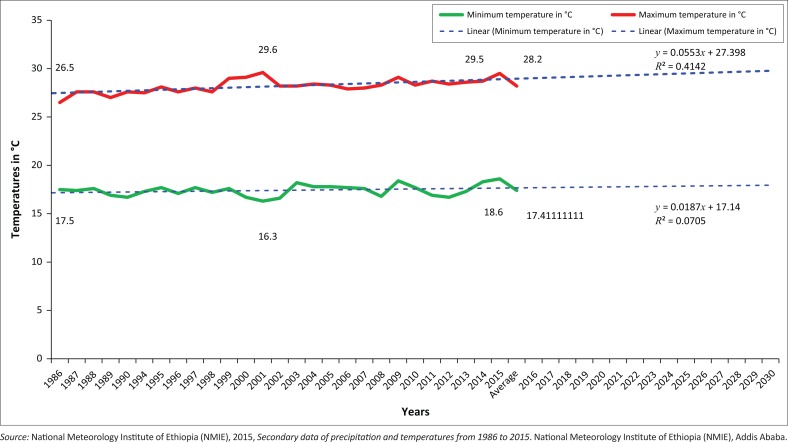
Konso annual average maximum and minimum temperatures (°C) for period 1986-2015 and projection up to 2050.

The Konso people have been known for their intensive agricultural practice and experience of indigenous knowledge in the field of natural environment conservation for more than 400 years (Watson 2009 cited in Demeulenaere 2010:322–325). Their cultural landscape was registered as a World Heritage site (Beyene 2007:48). Agriculture is the dominant economic activity in the study area. However, the agricultural production system is mainly dependent on rainfall in the area. The last three decades’ meteorological reports reveal that irregular seasonal and annual rainfall distribution was observed in the study site (Tadesse 2010:29). Although prediction of future rainfall is hampered by the El-Niño Southern Oscillation, drought and extreme weather events are likely to occur more often (UN-Habitat 2014:160). Precipitation and temperature data captured from National Meteorological Institute of Ethiopia (NMIE 2015) are depicted in Figures 2 and 3, respectively.

The findings reveal that over 80% of the respondents recognise the severity of potential climate-change-induced risks such as drought, shortages of water and food, reduced agricultural productivity, the prevalence of weather-related tropical diseases and heat waves, because of increasing temperature in the study area. However, the risks of landslides, soil erosion and loss of grazing land were rated low.

Incomes in Karat are low, with three-quarters of the population earning under R2000 per month (Author field survey 2015). Limited income-generation opportunities, coupled with a high unemployment rate and thus dependency ratio, increase households’ vulnerability. There is a small-scale traditional production system in the form of weaving, blacksmithing and handcrafts, which helps some of the community members to generate an additional income. Furthermore, the community employs a traditional saving and credit system ‘Equb’, and a government-sponsored safety net programme helps them to build assets to improve their resilience (Author survey 2016).

The outcome of target group discussion reveals that the existence of a strong long-standing social network creating social capital to conserve the natural environment helps them to survive in a hostile environment. Traditional leaders mobilise the community to conserve the natural environment. These cultural landscapes and the society’s traditions attract many tourists to the area, although there are limited infrastructure facilities in the area. The community also tries to adapt to periodic droughts through planting drought-resistant trees and a mixed cropping system. In addition, the traditional and cultural housing is made from materials that have good thermal insulation properties.

The people in the Konso area are well known for their long-time indigenous knowledge systems in natural environment conservation, terracing, mixed agricultural system, traditional medicines, traditional weather forecasting and other practices (Tadesse 2010:32). However, the investigation revealed that this knowledge system was not appropriately documented nor is it being transferred to the younger generation. Therefore, its application is limited to the elderly and subject to loss. A lack of institutional capacity at local level to integrate the indigenous knowledge with scientific knowledge also hampers the potential benefit that could be gained to cope with climate change risks in the area. The urban planning approach in the study area is strongly influenced by formal procedures and standards suggested by western planning theories and approaches. Such a top–down approach dominates the planning process that does not facilitate adaptive management to augment local resilience.

### Resilience to climate change

The resilience to climate change risks of the residents of Phuthaditjhaba and Konso, respectively, based on the capitals available to them, was calculated as explained in Method section. The higher the value, the higher the resilience. Thus, values below 0.05 indicate low resilience capacity.

According to the calculated risk index, both study areas have a low resilience because of the low levels of capitals available. This increases their vulnerability to risks including that of climate change. Phuthaditjhaba scored slightly better than Karat based on the indicators adopted.

### Ethical considerations

This letter confirms that a research proposal with tracking number ‘UFS-HSD2015/0023’ and title ‘Planning for Improving Resilience of Informal Settlements in the Mountainous Regions of Africa: Comparative Case Studies in QwaQwa-Phuthaditjhaba in South Africa; and Konso-Karat in Ethiopia’ was given ethical clearance by the Ethics Committee of the Faculty of Natural and Agricultural Sciences, University of the Free State.

## Discussion and conclusion

This comparative study identifies the unique environmental, spatial, social and economic features that determine the resilience capacity of both the case study areas. In both areas, all respondents identified a very high risk of droughts and water shortages and heat waves or hot summers that is commensurate with the predicted effects of climate change. The impacts of climate change on agriculture and the environment are also reflected in the perceptions of risk, although differently in each area. The long tradition of environmental management and protection of the soil through terracing in Karat is evident in the low perceived risks of soil erosion, loss of grazing land, flash flooding and rockfalls or landslides. Karat has higher levels of social capital, and this study suggests that through the practice of indigenous knowledge, their natural capital is better protected, thus reducing some of the risks under their control. The loss of traditional knowledge and weaker institutions of Phuthaditjhaba may contribute to the greater number of perceived environmental risks facing that community. Sadly, in both the study areas, the existing traditional knowledge is largely limited to the elders rather than being passed on to the younger generations.

The economic capital of both areas is low with over half of all households earning less than R2000 a month. Barua et al. (2014:267) found that, as in this study, indigenous knowledge provides coping mechanisms, but the communities’ livelihood options play an important role in their resilience to climate change. However, they also note that many factors, including access to health, education and ‘the ability to be heard’ (Barua et al. 2014:268), weaken their resilience. It can be argued that the Maluti-a-Phofung Municipality does not ‘hear’ the Phuthaditjhaba community in respect of physical capital.

The findings from the survey of community perceptions are at odds with the calculated climate change resilience index. This index, based on quantitative measurements of the number extent of infrastructure, institutions or indigenous knowledge, is not an actual reflection of the quality of the knowledge, the institutions or the infrastructure. It appears that the nature and the quality do make a difference. Furthermore, Quinlan et al. (2016:683) remark that by simplifying resilience measurements, the rich dynamics of the interactions of the SES can be lost.

The study also confirms the need to take a systemic view of local areas within the broader SES as advocated by De Bruijn et al. (2017:23). Consequently, the complex interactions between formal urban system, their peripheral informal settlements and rural areas must be acknowledged. In traditional communities, these systems are often part of a single SES. Hence, the impacts of climate change-induced risks in the areas of informal settlements may have a direct adverse effect on the formal urban system, because of these complex interrelations.

Given the potential of indigenous knowledge to promote climate change resilience capacity of communities, its role in climate change policies needs to be recognised. This is particularly important as many local governments, as in the case study areas, have limited capacity to cope with complex climate-change-related risks. Indigenous knowledge may facilitate and supplement the collective response to disaster risks and fill the gap that cannot be done otherwise. However, indigenous knowledge alone is not sufficient to ensure adequate livelihoods, especially when climate change is eroding the basis of those livelihoods and depriving communities of vital resources such as water.
